# Supplementary material for: Targeted computational design of an interleukin-7 superkine with enhanced folding efficiency and immunotherapeutic efficacy
Source: eLife. 2026 Jan 16;14:RP107671. doi: 10.7554/eLife.107671 (PMC12810954; doi:10.7554/eLife.107671)
Supplement: Figure 7—source data 1. [file elife-107671-fig7-data1.docx]

|  | **CD45+ CD3+** | **CD3+ CD4+** | **CD3+ CD8+** | **CD11C+ DC** | **CD19+ B-CELL** | **F480+ Macrophage** | **Ly6G- CD11b+ Monocyte** | **Ly6G+ CD11b+ NP** | **CD3+ CD4+ FoxP3+** | **CD3+ CD4+ PD1+** | **CD3+ CD4+ Perforin+** | **CD3+ CD8+ PD1+** | **CD3+ CD8+ Perforin+** |
| --- | --- | --- | --- | --- | --- | --- | --- | --- | --- | --- | --- | --- | --- |
| **FC-ctrl 1** | 485.87 | 31.32 | 62.65 | 24.39 | 147.19 | 285.48 | 103.95 | 40.35 | 44.77 | 21.84 | 1.91 | 17.50 | 10.03 |
| **FC-ctrl 2** | 53732 | 49.46 | 46.25 | 36.68 | 34.52 | 324.37 | 184.01 | 122.93 | 8.42 | 13.11 | 1.00 | 3.82 | 1.04 |
| **FC-ctrl 3** | 426.80 | 23.54 | 39.48 | 65.04 | 33.04 | 327.09 | 246.05 | 73.82 | 2.49 | 3.85 | 0.49 | 7.02 | 1.37 |
| **FC-ctrl 4** | 447.41 | 41.98 | 35.31 | 77.28 | 61.48 | 320.74 | 238.52 | 91.85 | 17.78 | 22.04 | 1.48 | 23.89 | 15.37 |
| **WT-IL7-1** | 812.81 | 51.36 | 265.97 | 64.12 | 42.96 | 344.99 | 267.30 | 100.41 | 5.96 | 5.70 | 1.48 | 8.17 | 2.30 |
| **WT-IL7-2** | 880.28 | 41.26 | 269.61 | 71.58 | 55.02 | 490.11 | 254.88 | 100.35 | 16.00 | 10.42 | 2.95 | 142.53 | 79.26 |
| **WT-IL7-3** | 1347.39 | 74.23 | 498.74 | 131.17 | 42.52 | 410.81 | 271.35 | 40.00 | 23.51 | 14.32 | 1.35 | 14.32 | 2.16 |
| **WT-IL7-4** | 442.51 | 58.49 | 190.61 | 22.54 | 24.97 | 99.26 | 52.43 | 56.35 | 8.33 | 2.82 | 0.18 | 6.79 | 1.35 |
| **N7-Q6P-1** | 5693.51 | 132.07 | 3424.14 | 64.68 | 190.27 | 721.44 | 178.02 | 34.23 | 29.32 | 11.89 | 0.68 | 117.70 | 54.32 |
| **N7-Q6P-2** | 6294.12 | 152.35 | 3695.88 | 76.27 | 201.37 | 590.59 | 181.96 | 151.96 | 10.59 | 14.85 | 0.74 | 14.71 | 8.09 |
| **N7-Q6P-3** | 3435.45 | 126.18 | 2463.09 | 46.67 | 325.04 | 305.20 | 153.98 | 48.78 | 14.88 | 16.22 | 2.07 | 889.39 | 364.63 |
| **N7-Q6P-4** | 6236.99 | 182.80 | 4060.65 | 118.28 | 327.31 | 1071.40 | 303.23 | 54.62 | 46.13 | 45.81 | 1.29 | 1117.42 | 452.26 |
| **N7-Q6PT45I-1** | 9662.56 | 167.86 | 6798.29 | 101.54 | 295.04 | 724.79 | 223.59 | 33.85 | 36.92 | 36.41 | 5.13 | 1090.51 | 333.85 |
| **N7-Q6PT45I-2** | 6205.21 | 43.56 | 5590.60 | 39.24 | 98.16 | 299.68 | 105.65 | 31.24 | 6.95 | 8.76 | 0.57 | 85.43 | 122.29 |
| **N7-Q6PT45I-3** | 5110.91 | 60.00 | 2251.52 | 133.33 | 164.24 | 564.24 | 236.36 | 84.85 | 29.09 | 12.73 | 0.45 | 799.09 | 510.00 |
| **N7-Q6PT45I-4** | 5870.48 | 193.74 | 4328.44 | 58.23 | 110.75 | 397.28 | 117.82 | 40.27 | 48.98 | 25.71 | 1.84 | 499.80 | 202.24 |

**Figure 7-source data 1.** Absolute count of immune cells determined from TILs/mg of the tumor excised
